# Assessment of Personality Profiles Among Patients Presenting With Deliberate Self-Harm at a Tertiary Care Hospital: A Cross-Sectional Study

**DOI:** 10.7759/cureus.105519

**Published:** 2026-03-19

**Authors:** Stuti J Mashru, Mohan Reddy Matti, Ruth Sneha Chandrakumar

**Affiliations:** 1 Psychiatry, Sri Devaraj Urs Academy of Higher Education and Research, Kolar, IND

**Keywords:** deliberate self-harm, international personality disorder examination, personality disorder, self-poisoning, socioeconomic factors, suicide

## Abstract

Background

Deliberate self-harm (DSH) represents intentional self-inflicted physical injury encompassing both non-suicidal self-injurious acts and suicide attempts, constituting a significant psychiatric emergency with profound morbidity implications. Individuals with personality disorders demonstrate substantially elevated suicide attempt rates compared to those without personality pathology. Comprehensive psychosocial assessment remains fundamental for evaluating DSH presentations. While research has investigated socioeconomic determinants and personality disorders among adolescent populations, investigations examining general adult populations remain limited. This study determined personality profiles among DSH patients and investigated relationships between sociodemographic factors and DSH behaviors.

Methodology

This analytical cross-sectional investigation enrolled 193 adults presenting with DSH to the RL Jalappa Hospital Psychiatry Department, Sri Devaraj Urs Medical College, Kolar, Karnataka, India, between September 2022 and December 2023. Comprehensive histories were obtained through patient interviews and reliable informant collateral and discussed with departmental psychiatrists. Personality diagnoses were established according to the International Classification of Diseases, 10th Revision (ICD-10) and the Diagnostic and Statistical Manual of Mental Disorders, Fifth Edition (DSM-5) criteria using the International Personality Disorder Examination screening questionnaire, with responses documented and scored.

Results

Among 193 participants, the mean age was 31 years (31.46±9.75), with 99 females (51.3%) and 94 males (48.7%). Lower socioeconomic status characterized 70% (n=136), while 132 were unmarried (68.4%). Ninety-three participants were unemployed (48.2%), with 164 from rural areas (84.9%). One hundred eleven participants (57.5%) attempted DSH without psychiatric illness, whereas 82 (42.5%) had psychiatric comorbidity: adjustment disorders, 33 cases (17.1%), depression, 22 cases (11.4%). One hundred and three participants (53.4%) reported previous suicide attempts. Family conflicts precipitated stress (n=60, 60.6%) and financial difficulties (n=22, 22.2%). Methods included liquid poison in 127 cases (65.8%), multiple tablets in 28 (14.5%), hanging in 24 (12.4%), and self-cutting in 15 (7.8%). Substance abuse affected 97 participants (50.25%). Borderline personality predominated in 48 cases (24.9%), dependent in 42 (21.8%), and impulsive in 27 (14%). Cluster B constituted 98 cases (50.8%), Cluster C 66 (34.2%), and Cluster A 29 (15%). Significant Cluster B associations included younger age, female gender, lower education, lower socioeconomic status, unmarried status, unemployment, recurrent attempts, stressors, substance abuse, and the liquid poison method (p<0.05).

Conclusion

Psychiatric disorders constitute established self-harm risk factors, with findings demonstrating literature consistency. Cluster B personality disorders, particularly borderline traits, predominate among DSH presentations. Multiple sociodemographic vulnerabilities, including younger age, female gender, socioeconomic disadvantage, unemployment, and substance abuse, are significantly associated with personality clustering. Larger investigations warrant a comprehensive personality profile evaluation among suicide attempters.

## Introduction

Suicide constitutes a significant global public health concern, accounting for approximately 1.5% of annual mortality worldwide [1]. The identification of factors associated with suicidal behavior remains critical for risk stratification and targeted intervention strategies [2]. Among established risk factors, prior suicide attempts represent the most robust predictor of eventual death by suicide, with individuals who have previously attempted suicide demonstrating substantially elevated risk compared to those without such a history [3]. Furthermore, suicide attempts predict diverse adverse outcomes, including physical health deterioration, mental disorders, interpersonal difficulties, and increased healthcare utilization [3,4].

Deliberate self-harm (DSH) refers to intentional acts of self-inflicted physical injury undertaken without lethal intent [5]. Common manifestations include cutting with sharp implements, self-striking, intentional drug overdose, food intake restriction, and engagement in risk-taking behaviors [6]. Although DSH behaviors are typically performed without suicidal intention, they may inadvertently result in fatality [5]. Personality characteristics fundamentally determine individual responses to stressful circumstances [7]. Individuals with personality disorders experience distorted reality perception and abnormal affective responses, manifesting as pervasive distress across occupational, social, and interpersonal domains [8]. These personality disorders represent chronic, maladaptive patterns of behavior, cognition, and mood, constituting established risk factors for both fatal and nonfatal suicidal behaviors [9].

Research demonstrates that DSH subjects with comorbid personality disorders exhibit significantly higher suicide attempt rates compared to those without such disorders [10]. Most patients report experiencing extreme tension, anxiety, anger, or fear preceding self-harm episodes, with the behavior positively reinforced through subsequent relief, satisfaction, and decreased tension [11]. In the Indian context, limited attention has been directed toward individuals experiencing self-harm or associated risk behaviors [12,13]. Socioeconomic inequality has contributed to increased self-inflicted harm among vulnerable populations, with social and cultural norms playing substantial roles in shaping behavioral patterns [14].

Psychosocial assessment represents a crucial domain for DSH evaluation. Although several investigations have examined socioeconomic factors associated with DSH among adolescent populations, limited research exists regarding personality profiles in general adult populations. The present study was therefore undertaken to determine the personality profile of adult patients with DSH history and to identify associated sociodemographic factors influencing these presentations. Based on existing literature, we hypothesized that Cluster B personality disorders, particularly borderline traits, would predominate among DSH presentations, and that specific sociodemographic vulnerabilities, including younger age, female gender, lower socioeconomic status, and substance abuse, would demonstrate significant associations with Cluster B personality clustering. In rural South Indian contexts, DSH presentations are distinctively shaped by agricultural pesticide accessibility, joint family interpersonal conflicts, dowry-related psychosocial stressors, limited mental health literacy, and entrenched socioeconomic disadvantage. These culturally embedded determinants necessitate India-specific epidemiological investigation rather than uncritical extrapolation from Western or urban psychiatric literature.

## Materials and methods

Study design and study setting

This analytical cross-sectional investigation was conducted to determine personality profiles among patients with DSH and to examine relationships between sociodemographic factors and DSH behaviors. The study was undertaken at RL Jalappa Hospital, a teaching institution affiliated with Sri Devaraj Urs Medical College, a constituent college of Sri Devaraj Urs Academy of Higher Education and Research, located in Kolar, Karnataka, India. All patients presenting to the psychiatry department with a DSH history during the study period were eligible for enrollment, irrespective of subsequent admission or discharge status, provided they satisfied the stipulated inclusion criteria and provided informed consent.

Study period

The study enrollment and data collection spanned 16 months, from September 2022 through December 2023, encompassing all consecutive DSH presentations during this timeframe.

Ethics committee approval

The Institutional Ethics Committee granted formal approval for this investigation under approval number SDUMC/KLR/IEC/285/2022-23. Researchers ensured participant confidentiality and privacy throughout the study, utilizing collected data exclusively for research purposes. Informed consent was obtained from all participants prior to enrollment.

Inclusion criteria

Patients aged 18 to 60 years presenting with DSH attempts and providing informed consent for screening questionnaire administration were included in the study.

Exclusion criteria

Patients with severe intellectual disability precluding comprehension of interview procedures, those suffering from severe mental disorders preventing meaningful participation, individuals with severe physical or neurological disorders, and patients previously diagnosed with personality disorders were excluded from this investigation. "Severe mental disorders preventing meaningful participation" meant patients who were experiencing active psychosis, serious dissociative states, or major cognitive issues that made it hard for them to respond clearly during interviews, based on the consulting psychiatrist's assessment when they were admitted. Patients with depression, adjustment disorders, and anxiety conditions highly prevalent in DSH populations, were explicitly retained.

Sample size estimation

Sample size calculation employed Cochran's formula: $n = \frac{Z^2 \times p \times (1-p)}{d^2}$ where Z represents the standard normal variate, p denotes prevalence, and d indicates precision. Saini et al. (2021) conducted a cross-sectional study among 525 psychiatric patients in a tertiary hospital over one year, reporting personality disorder trait prevalence of 56.3% among psychiatric patients [15]. Utilizing this prevalence estimate (p=0.563), 95% confidence level (Z=1.96), and 7% absolute precision (d=0.07), the calculation yielded:

\(n = \frac{(1.96)^2 \times 0.563 \times (1 - 0.563)}{(0.07)^2} = \frac{3.8416 \times 0.563 \times 0.437}{0.0049} = 192.76\), rounded to 193 participants.

Therefore, the minimum required sample size was determined to be 193 participants.

Sampling method

Consecutive convenience sampling was employed, enrolling all patients diagnosed with DSH and admitted to the psychiatry department of RL Jalappa Hospital between September 2022 and December 2023 who satisfied the inclusion criteria. Findings should be interpreted with the recognition that tertiary care sampling may over-represent severe pathology, potentially limiting generalizability to community-dwelling or primary care DSH populations.

Data collection procedure

Comprehensive DSH histories were obtained through structured interviews with patients and corroborative information from reliable informants. Detailed sociodemographic data, including age, gender, educational attainment, socioeconomic status, marital status, occupational status, residential location, religious affiliation, birth order, and family type, were systematically documented. Clinical variables encompassing psychiatric diagnosis, previous suicide attempt history, number of suicide attempts, presence of psychosocial stressors, stressor categorization, substance abuse patterns, medical comorbidities, and suicide attempt methodology were comprehensively recorded. Throughout this manuscript, "deliberate self-harm" denotes the inclusive clinical construct encompassing both non-suicidal self-injurious acts and suicide attempts, while "suicide attempt history" specifically refers to previously documented episodes with discernible suicidal intent, as established through a structured clinical interview. Suicidal intent was systematically assessed through structured psychiatric interviews evaluating stated intent, circumstances of the act, rescue factors, and post-attempt communication, with DSH classification assigned only when psychiatrists confirmed the absence of unambiguous lethal intent, irrespective of the method employed.

Each case was subsequently discussed with departmental psychiatrists to ensure diagnostic accuracy. Diagnostic consensus was achieved through structured case discussion with departmental psychiatrists; cases presenting diagnostic ambiguity regarding intent classification or personality profiling were resolved through multidisciplinary consensus review rather than single-clinician determination. Collateral informant histories served a corroborative function, supplementing patient self-report, particularly in cases where acute distress, cognitive impairment, or suspected underreporting warranted independent verification of clinical variables.

Personality profile diagnoses were established according to the International Classification of Diseases, 10th Revision (ICD-10) and the Diagnostic and Statistical Manual of Mental Disorders, Fifth Edition (DSM-5) criteria [16]. The International Personality Disorder Examination (IPDE) screening questionnaire, a validated self-administered instrument requiring 10 to 15 minutes of completion time, was utilized for personality disorder assessment [17,18]. This questionnaire has demonstrated reliability and validity across multiple international investigations for identifying personality disorder traits and cluster classifications. Patients responded True or False to questionnaire items, with responses systematically scored to identify specific personality disorders and cluster groupings (Cluster A, Cluster B, and Cluster C). Patients scoring positively for predetermined thresholds in any personality disorder criteria were classified as having that personality disorder. All IPDE screening-positive cases were clinically reviewed and confirmed by departmental psychiatrists before final personality disorder classification was assigned.

Data analysis

Collected data were imported into Microsoft Excel (Microsoft Corp., Redmond, WA, USA) and analyzed using IBM SPSS Statistics for Windows, Version 26 (Released 2018; IBM Corp., Armonk, New York, United States). Descriptive statistics employed frequency analysis and percentage calculations for discrete variables, while continuous variables were characterized using mean, median, and standard deviation. Inferential statistical analysis utilized the chi-square test or Fisher's exact test for examining statistically significant differences in discrete variables between groups and the independent t-test for continuous variable comparisons. The statistical significance threshold was established at a probability value of 0.05 for all analytical procedures.

## Results

The baseline sociodemographic profile in Table 1 demonstrated a mean age of 31.46 years with a standard deviation of 9.75 years. Gender distribution revealed a marginal female preponderance with 99 participants (51.30%) compared to 94 male participants (48.70%). Socioeconomic stratification indicated substantially lower-income representation among 136 participants (70.47%), while marital status analysis revealed unmarried predominance among 132 participants (68.39%). Geographic distribution demonstrated predominantly rural residence among 164 participants (84.97%), with educational attainment concentrated at the secondary school level among 70 participants (36.27%). Clinical characteristics established that 103 participants (53.4%) had a previous suicide attempt history, while psychosocial stressors were identified among 99 participants (51.30%). Substance abuse patterns were documented among 97 participants (50.26%), and medical comorbidities were present among 48 participants (24.87%), collectively establishing a comprehensive demographic and clinical baseline profile characteristic of DSH populations in rural South Indian tertiary care settings.

**Table 1:** Baseline sociodemographic and clinical characteristics of study participants with deliberate self-harm (N=193) Data are presented as mean ± standard deviation (SD) for continuous variables and frequency (percentage) for categorical variables.

Characteristic	n (%) or mean ± SD
Age (years)	31.46 ± 9.75 (Range: 18-60)
Gender	
Female	99 (51.30)
Male	94 (48.70)
Socioeconomic Status	
Lower income	136 (70.47)
Middle income	57 (29.53)
Marital Status	
Unmarried	132 (68.39)
Married	61 (31.61)
Occupational Status	
Employed	100 (51.81)
Unemployed	93 (48.19)
Place of Residence	
Rural	164 (84.97)
Urban	29 (15.03)
Educational Level	
Secondary school	70 (36.27)
High school	63 (32.64)
Graduate	38 (19.69)
Primary school	12 (6.22)
Uneducated	10 (5.18)
Religious Affiliation	
Hindu	184 (95.30)
Muslim	9 (4.70)
Previous Suicide Attempts	103 (53.4)
Presence of Psychosocial Stressor	99 (51.30)
Substance Abuse	97 (50.26)
Medical Comorbidities	48 (24.87)

The psychiatric diagnostic distribution in Table 2 revealed that 111 participants (57.50%) engaged in DSH without concurrent psychiatric illness, establishing a substantial proportion of behaviorally mediated self-harm independent of formal psychiatric diagnosis. Psychiatric diagnoses were established through structured psychiatrist-administered clinical interviews per ICD-10/DSM-5 criteria. Among those with psychiatric comorbidity, adjustment disorders represented the predominant diagnosis among 33 participants (17.10%), followed by major depressive disorder among 22 participants (11.40%) and acute stress reactions among 10 participants (5.20%). Psychosocial stressor analysis among the 99 participants reporting stress identified family conflict as the predominant precipitant among 60 participants (60.60%), with financial difficulties constituting the secondary stressor among 22 participants (22.20%). Methodological examination of self-harm approaches demonstrated liquid poison ingestion as the predominant method among 126 participants (65.28%), consistent with agricultural pesticide accessibility in rural settings, followed by multiple tablets among 28 participants (14.51%). Substance abuse patterns revealed alcohol consumption as the primary substance among 32 participants (16.58%) using alcohol exclusively, with polysubstance use combinations observed among an additional 32 participants (16.58%). Medical comorbidity assessment identified hypertension as the most prevalent condition among 17 participants (8.81%), followed by seizure disorders among 12 participants (6.22%), collectively establishing a comprehensive psychiatric, behavioral, and medical characterization of the study population.

**Table 2:** Psychiatric diagnosis distribution and method of deliberate self-harm (DSH) among study participants (N=193) Data are presented as frequency (percentage).

Variable	n (%)
Psychiatric Diagnosis	
DSH without psychiatric illness	111 (57.50)
DSH with adjustment disorders	33 (17.10)
DSH with depression	22 (11.40)
DSH with acute stress reaction	10 (5.20)
DSH with panic disorder	6 (3.10)
DSH with dysthymic disorders	5 (2.60)
DSH with anxiety disorder	3 (1.60)
DSH with grief reaction	3 (1.60)
Primary Stressor Type (n=99)	
Family issues	60 (60.60)
Financial issues	22 (22.20)
Spousal conflict	6 (6.10)
Somatic complaints	6 (6.10)
Extramarital affair	5 (5.10)
Method of Suicide Attempt	
Liquid poison ingestion	126 (65.28)
Multiple tablets	28 (14.51)
Hanging	24 (12.44)
Self-cutting	15 (7.77)
Substance Abuse Pattern	
Alcohol alone	32 (16.58)
Alcohol and smoking	18 (9.33)
Alcohol and tobacco	15 (7.77)
Other combinations	32 (16.58)
Medical Comorbidity Distribution	
Hypertension	17 (8.81)
Seizure disorder	12 (6.22)
Diabetes mellitus	11 (5.70)
Other conditions	8 (4.15)

Personality trait assessment utilizing the IPDE screening questionnaire in Table 3 revealed borderline personality characteristics as the predominant trait among 48 participants (24.87%), characterized by emotional dysregulation, impulsivity, and unstable interpersonal relationships. Dependent personality features constituted the second most prevalent trait among 42 participants (21.76%), manifesting as an excessive need for nurturance and submissive behavior patterns. Impulsive personality characteristics were identified among 27 participants (13.99%), demonstrating poor impulse control and behavioral disinhibition. According to DSM-5 cluster classification, Cluster B personality disorders, encompassing dramatic, emotional, and erratic behavioral patterns, demonstrated the highest prevalence among 98 participants (50.78%), representing more than half of the study cohort. Cluster C anxious and fearful personality patterns were documented among 66 participants (34.20%), while Cluster A odd and eccentric personality characteristics were observed among 29 participants (15.02%). This distribution pattern aligns with established literature documenting the predominance of Cluster B personality disorders among individuals engaging in DSH behaviors, with borderline personality disorder representing the archetypal association with recurrent self-injurious behaviors and emotional dysregulation patterns observed in clinical psychiatric populations worldwide.

**Table 3:** Personality trait and cluster distribution according to the International Personality Disorder Examination (IPDE) (N=193) Personality assessment conducted using the IPDE screening questionnaire according to ICD-10 and DSM-5 diagnostic criteria. Cluster A includes paranoid and schizoid personalities; Cluster B includes borderline, histrionic, impulsive, and dissocial personalities; and Cluster C includes anxious, dependent, and anankastic personalities. Data are presented as frequency (percentage). ICD-10: International Classification of Diseases, 10th Revision; DSM-5: Diagnostic and Statistical Manual of Mental Disorders, Fifth Edition

Personality Classification	n (%)
Individual Personality Traits	
Borderline	48 (24.87)
Dependent	42 (21.76)
Impulsive	27 (13.99)
Anxious	18 (9.33)
Schizoid	16 (8.29)
Histrionic	14 (7.25)
Paranoid	13 (6.74)
Dissocial	9 (4.67)
Anankastic	6 (3.10)
DSM-5 Personality Cluster Classification	
Cluster B (Dramatic/Erratic)	98 (50.78)
Cluster C (Anxious/Fearful)	66 (34.20)
Cluster A (Odd/Eccentric)	29 (15.02)

Univariate analysis in Table 4 examining sociodemographic associations with personality cluster classifications revealed highly significant age-related stratification (χ^2^=28.111, p<0.001), with Cluster B demonstrating marked predominance among younger adults aged 18-30 years, comprising 78 participants (59.54%) compared to nine participants (6.87%) in Cluster A. Gender distribution established statistically significant variation (χ^2^=7.858, p=0.020), with female participants demonstrating Cluster B preponderance among 60 participants (60.61%) versus 12 participants (12.12%) in Cluster A. Socio//economic stratification revealed a highly significant association (χ^2^=9.712, p=0.007), with lower-income groups manifesting Cluster B characteristics among 78 participants (57.35%). Marital status demonstrated the most robust association (χ^2^=13.904, p=0.001), with unmarried individuals exhibiting Cluster B predominance among 79 participants (59.85%), contrasted with married individuals showing more equitable cluster distribution. Educational attainment revealed significant clustering (χ^2^=9.924, p=0.041), with school-educated participants predominantly exhibiting Cluster B traits among 82 participants (56.55%). Employment status demonstrated a significant association (χ^2^=6.495, p=0.038), with unemployed participants showing Cluster B preponderance among 56 participants (60.22%). Geographic residence, however, demonstrated no statistically significant association with personality clustering (χ^2^=0.870, p=0.647), suggesting that urbanization status does not substantially influence personality disorder phenotypic expression in DSH populations, thereby establishing that individual sociodemographic characteristics rather than geographic factors predominantly determine personality cluster distribution patterns.

**Table 4:** Univariate analysis of sociodemographic factors associated with personality cluster classification among patients with deliberate self-harm Cluster A: Odd/Eccentric personalities; Cluster B: Dramatic/erratic personalities; Cluster C: Anxious/fearful personalities. Data were presented as frequency (percentage). Statistical analysis was performed using the chi-square test, and the statistical significance threshold was established at a p-value less than 0.05.

Sociodemographic Variable	Cluster A (n=29)	Cluster B (n=98)	Cluster C (n=66)	χ^2^ value	p-value
Age Group (years)					
18-30	9 (6.87)	78 (59.54)	44 (33.59)	28.111	0.001
31-45	13 (33.33)	9 (23.08)	17 (43.59)		
46-60	7 (30.43)	11 (47.83)	5 (21.74)		
Gender					
Female	12 (12.12)	60 (60.61)	27 (27.27)	7.858	0.020
Male	17 (18.09)	38 (40.43)	39 (41.49)		
Educational Status					
Uneducated	3 (30.00)	4 (40.00)	3 (30.00)	9.924	0.041
School education	17 (11.72)	82 (56.55)	46 (31.72)		
Graduate	9 (23.68)	12 (31.58)	17 (44.74)		
Socioeconomic Status					
Lower income	15 (11.03)	78 (57.35)	43 (31.62)	9.712	0.007
Middle income	14 (24.56)	20 (35.09)	23 (40.35)		
Marital Status					
Unmarried	17 (12.88)	79 (59.85)	36 (27.27)	13.904	0.001
Married	12 (19.67)	19 (31.15)	30 (49.18)		
Occupational Status					
Unemployed	12 (12.90)	56 (60.22)	25 (26.88)	6.495	0.038
Employed	17 (17.00)	42 (42.00)	41 (41.00)		
Place of Residence					
Rural	23 (14.02)	84 (51.22)	57 (34.76)	0.870	0.647
Urban	6 (20.69)	14 (48.28)	9 (31.03)		

Univariate clinical variable analysis in Table 5 established multiple statistically significant associations with personality cluster distribution. Previous suicide attempt history demonstrated significant clustering (χ^2^=6.070, p=0.048), with Cluster B exhibiting predominance among 59 participants (59.00%) with prior attempts. The frequency of suicide attempts revealed progressive Cluster B escalation, with 31 participants (72.10%) having three or more attempts compared to two participants (4.70%) in Cluster A (χ^2^=11.59, p=0.02), establishing a dose-response relationship between attempt frequency and Cluster B personality characteristics. Psychosocial stressor presence demonstrated a highly significant association (χ^2^=13.650, p=0.001), with 63 participants (63.64%) reporting stressors manifesting Cluster B traits. Substance abuse patterns revealed highly significant clustering (χ^2^=11.441, p=0.003), with 61 participants (62.89%) engaging in substance use demonstrating Cluster B characteristics. Methodological analysis of self-harm approaches demonstrated significant heterogeneity (χ^2^=13.899, p=0.030), with liquid poison ingestion predominating in Cluster B among 72 participants (57.14%), while hanging and self-cutting methods demonstrated Cluster C preponderance among 12 participants (50.00%) and nine participants (60.00%), respectively, suggesting distinct behavioral phenotypes across personality classifications. Medical comorbidity presence, birth order, and family structural configuration demonstrated no statistically significant associations, with p-values exceeding 0.05, thereby establishing that behavioral and psychological variables rather than structural or biological factors predominantly influence personality cluster distribution in DSH populations.

**Table 5:** Univariate analysis of clinical and behavioral factors associated with personality cluster classification Data were presented as frequency (percentage). Statistical analysis was performed using the chi-square test, and the statistical significance threshold was established at a p-value less than 0.05.

Clinical Variable	Cluster A (n=29)	Cluster B (n=98)	Cluster C (n=66)	χ^2^ value	p-value
Previous Suicide Attempts					
Yes	11 (11.00)	59 (59.00)	30 (30.00)	6.070	0.048
No	18 (19.35)	39 (41.94)	36 (38.71)		
Number of Suicide Attempts					
None	16 (17.78)	38 (42.22)	36 (40.00)	11.59	0.02
Less than 3	11 (18.33)	29 (48.33)	20 (33.30)		
Three or more	2 (4.70)	31 (72.10)	10 (23.30)		
Presence of Psychosocial Stressor					
Yes	12 (12.12)	63 (63.64)	24 (24.24)	13.650	0.001
No	17 (18.09)	35 (37.23)	42 (44.68)		
Substance Abuse					
Yes	11 (11.34)	61 (62.89)	25 (25.77)	11.441	0.003
No	18 (18.75)	37 (38.54)	41 (42.71)		
Method of Suicide Attempt					
Liquid poison	16 (12.70)	72 (57.14)	38 (30.16)	13.899	0.030
Multiple tablets	8 (28.57)	13 (46.43)	7 (25.00)		
Hanging	3 (12.50)	9 (37.50)	12 (50.00)		
Self-cutting	2 (13.33)	4 (26.67)	9 (60.00)		
Medical Comorbidities					
Yes	11 (22.92)	24 (50.00)	13 (27.08)	3.160	0.165
No	18 (12.41)	74 (51.03)	53 (36.55)		
Birth Order					
≤2	23 (14.65)	83 (52.87)	51 (32.48)	5.807	0.831
>2	6 (16.67)	15 (41.67)	15 (41.67)		
Family Type					
Nuclear	23 (14.94)	80 (51.95)	51 (33.12)	0.470	0.791
Joint	6 (15.38)	18 (46.15)	15 (38.46)		

## Discussion

The present cross-sectional study investigated personality profiles among 193 adult patients with DSH presenting to a tertiary care hospital in rural Karnataka, revealing borderline personality traits as the predominant characteristic (48, 24.9%), and Cluster B personality trait clustering was identified at the highest frequency (98, 50.8%) among IPDE screening-positive cases. These findings align substantially with international literature documenting personality disorder predominance among DSH populations, though notable variations exist in prevalence rates and demographic associations across geographical and cultural contexts.

The psychiatric comorbidity prevalence of 42.5% (n=82) in our cohort, with adjustment disorders (33, 17.1%) and depression (22, 11.4%) as leading diagnoses, demonstrates consistency with previous Indian investigations. Sharma (1998) reported psychiatric disorders among 46.7% of suicide attempters, while Jain et al. (1999) documented psychiatric illness in 57% of their sample [19,20]. Our findings fall within this established range, though they are lower than the 93% psychiatric diagnosis rate reported by Latha et al. (1996) among 73 suicide attempters in India [21]. This variation likely reflects methodological differences in diagnostic assessment protocols and sample selection criteria across studies. Internationally, Manoranjitham et al. (2010) identified psychiatric diagnoses in 37% of suicide decedents in rural South India, with alcohol dependence (16%) and adjustment disorders (15%) as predominant categories, corroborating our adjustment disorder prevalence pattern [22].

The prevalence of personality disorders in our study warrants comparison with existing Indian literature. Previous investigations in India have documented personality disorder rates ranging from 7% to 50% among suicide attempters [21,23,24]. The most common diagnoses identified in prior research include schizoid, borderline, and antisocial personality disorders (Gupta et al., 1992), while Chandrasekaran et al. (2003) reported anankastic and histrionic personality disorders as predominant among first attempters [23,24]. Our findings establishing borderline personality disorder predominance (48, 24.9%) align more closely with Western literature patterns, potentially reflecting evolving diagnostic practices and increased recognition of borderline personality characteristics in Indian psychiatric settings.

Previous suicide attempt history, documented among 53.4% (n=103) of our cohort, corresponds with established literature identifying prior attempts as the most robust predictor of future suicidal behavior. Khan et al. (2005) observed previous attempt history in 16% of their sample, substantially lower than our finding [25]. This discrepancy may reflect our tertiary care setting attracting more severe, recurrent cases compared to community-based samples. A progressive frequency pattern was observed between suicide attempt recurrence and Cluster B personality trait classification - with 72.1% (n=31) of individuals with three or more attempts demonstrating Cluster B characteristics - a finding consistent with international literature documenting borderline personality pathology associations with repetitive self-harm, though causal directionality cannot be established from this cross-sectional design.

Psychosocial stressor analysis revealed family conflict as the predominant precipitant (60, 60.6%), followed by financial difficulties (22, 22.2%), mirroring patterns documented across Indian studies. Sahoo et al. (2018) identified family conflicts, peer-interpersonal problems, and perceived humiliations as principal risk factors among 100 suicide attempters [26]. Mohanty et al. (2007) documented financial burden (37%) and marital disharmony (35%) as predominant reasons among 588 suicide victims autopsied, though their sample represented completed rather than attempted suicides [27]. The sociocentric nature of Indian society, emphasizing interpersonal relationships and family cohesion, renders family conflict particularly salient as a precipitating factor, as noted by Banerjee et al. (1990) in their examination of vulnerability factors among Indian women [28].

Methodological preferences for self-harm demonstrated liquid poison ingestion predominance (127, 65.8%), consistent with established patterns in rural Indian populations. Latha et al. (1996) documented poisoning via insecticides and pesticides in 67% of attempts, while Banerjee et al. (1990) and Khan et al. (2005) similarly identified poisoning as the predominant method [25,28]. The ready availability of agricultural pesticides in rural settings, combined with the impulsive decision-making characteristic of Cluster B personalities, explains this methodological predominance. Conversely, Western populations demonstrate different methodological patterns, with medication overdose and self-cutting more prevalent, reflecting differential access to lethal means across cultural contexts. The striking predominance of liquid poison ingestion in our cohort reflects the convergence of agricultural pesticide ubiquity in rural Karnataka, impulsive access-driven decision-making, and the absence of means restriction infrastructure. This culturally embedded pattern underscores the public health imperative of pesticide storage regulation and community-level means restriction as primary prevention strategies in rural South Indian contexts, consistent with WHO recommendations for lethal means counseling.

Substance abuse prevalence of 50.26% (n=97), primarily alcohol consumption (32, 16.58%), aligns with previous Indian investigations. Latha et al. (1996) documented alcohol consumption in 27% of attempters, while Shafii et al. (1985) reported substance abuse among 70% of child and adolescent suicide completers in the United States [21,29]. Khan et al. (2005) identified substance abuse in 18% of their Indian sample, while Unni and Mani (1996) documented 9.74% prevalence among suicidal ideators [25,30]. The variability across studies likely reflects methodological differences in substance abuse assessment and population characteristics, with our tertiary care setting potentially attracting more severe polysubstance abuse cases.

The sociodemographic associations identified in our study demonstrate both convergence and divergence with established literature. The mean age of 31.46 years, with younger adult predominance (18-30 years) among Cluster B personalities, aligns with National Crime Records Bureau (2016) data indicating 34.4% of suicides occurring between ages 15 and 29 years [31]. Female gender predominance (99, 51.3%) in our sample corresponds with findings from Kar (2010), Bhugra and Desai (2002), and Lingeswaran (2016), though it contrasts with some international literature demonstrating male predominance in completed suicides [32-34]. The hostile family environment, preexisting personality pathology, and dowry-related stresses documented by Gururaj et al. (2001) and Kumar (2004) may explain elevated female self-harm rates in Indian contexts [35,36].

Educational status patterns, with 32.6% (n=63) completing high school, correspond with National Crime Records Bureau (2016) documentation of increasing student suicides from 5.5% (2010) to 6.2% (2013) [31]. Radhakrishnan and Andrade (2012) noted that low intelligence confers a two- to three-fold increased suicide risk, potentially through diminished competitive capacity, lower socioeconomic attainment, and reduced stress-coping efficiency [37]. Socioeconomic vulnerability, with 70.5% (n=136) belonging to lower income strata and 48.2% (n=93) unemployed, aligns with Srivastava et al. (2004), who identified unemployment in 46% of Indian suicide attempters, while Latha et al. (1996) documented unemployment in half their sample [21,38]. The economic stress hypothesis, supported by Manoranjitham et al. (2010), suggests unemployment creates hopelessness, compounding psychological vulnerability among economically disadvantaged populations [22].

Marital status findings, with 68.4% (n=132) unmarried, align with Sharma et al. (1998), documenting 52% unmarried status among attempters, though they contrast with Western literature identifying marriage as protective against suicide [20]. Krug et al. (2002) noted that marriage's protective effect diminishes in developing countries, potentially reflecting arranged marriage practices, social pressure maintaining abusive relationships, and limited divorce accessibility in Indian contexts [39]. Kumar et al. (2006) similarly identified single status predominance among suicide attempters in South India [40].

The personality-specific behavioral phenotypes identified warrant emphasis. Cluster B personality classification was significantly associated with younger age, female gender, lower socioeconomic status, unemployment, high-frequency attempts, psychosocial stressors, substance abuse, and impulsive methods, while Cluster C personalities exhibited anxiety-driven, deliberative patterns with planned methodologies. These distinct profiles support Nath et al.'s (2008) observations regarding personality-determined stress responses and Krysinska et al.'s (2006) documentation of personality disorder associations with both fatal and nonfatal suicidal behaviors [7,9]. The findings underscore Klonsky's (2007) functional model, wherein self-harm serves emotional regulation purposes, with reported extreme tension, anxiety, anger, and fear preceding acts, subsequently relieved through self-injury [11].

Clinical significance

The identification of Cluster B personality disorder predominance among DSH patients (50.8%), particularly borderline traits (24.9%), holds substantial clinical implications for psychiatric practice in resource-limited settings. The documented associations between personality clustering and specific sociodemographic vulnerabilities - younger age, female gender, lower socioeconomic status, unemployment, and substance abuse - enable the development of targeted screening protocols for high-risk populations presenting to primary care and emergency departments. The observed association between suicide attempt frequency and Cluster B personality trait classification supports consideration of intensive crisis intervention and dialectical behavior therapy approaches for recurrent attempters, while Cluster C personalities may benefit from anxiety-focused cognitive-behavioral interventions, thereby supporting personality-informed risk stratification and treatment allocation strategies in tertiary psychiatric settings.

Strengths of the study

The present investigation demonstrates multiple methodological strengths enhancing scientific rigor and generalizability. The utilization of the standardized IPDE screening questionnaire according to ICD-10 and DSM-5 criteria ensured diagnostic reliability and international comparability. The comprehensive sample size of 193 participants, calculated based on established prevalence estimates with 7% precision, provided adequate statistical power for detecting meaningful associations. The inclusion of diverse sociodemographic and clinical variables enabled multifaceted risk factor examination. The tertiary care hospital setting facilitated access to detailed psychiatric assessment and collateral history from reliable informants, enhancing diagnostic accuracy. The study's focus on the general adult population rather than exclusively adolescent samples addresses an identified literature gap, while the rural South Indian setting provides valuable data from underrepresented geographical regions.

Limitations

Several methodological limitations warrant acknowledgment. The cross-sectional design precludes causal inference regarding temporal relationships between personality characteristics and DSH behaviors, necessitating longitudinal investigations to establish directional associations. The hospital-based convenience sampling strategy introduces potential selection bias, as individuals presenting to tertiary psychiatric facilities may represent more severe pathology compared to community-based DSH populations, limiting generalizability to non-treatment-seeking individuals. The absence of comparison groups comprising non-suicidal DSH individuals or psychiatric controls without a self-harm history constrains differential association analysis. Important variables, including bullying history, loneliness, anxiety severity, temporal circumstances of self-harm episodes, precipitating event nature, communication of suicidal intent, attempt lethality assessment, and rescue factors, were not systematically evaluated, potentially omitting relevant risk factor domains. The absence of multivariate logistic regression analysis precludes identification of independent predictors of Cluster B personality clustering while controlling for confounding sociodemographic variables, representing a constraint on the interpretive precision of reported associations. Long-term outcome monitoring and predictive utility assessment of personality-based risk stratification could not be conducted within the study timeframe, limiting clinical applicability validation. Furthermore, acute crisis states at the time of assessment may have transiently amplified personality trait expression, potentially inflating disorder prevalence estimates. Concurrently, self-report bias inherent to questionnaire-based personality screening in acute psychiatric settings represents an additional threat to internal validity, as psychological distress during acute presentations may systematically distort self-reported personality trait endorsement.

Recommendations

Future investigations should employ prospective longitudinal designs with larger, multi-center samples incorporating community-based populations to enhance generalizability and establish temporal precedence. Implementation of comprehensive assessment protocols, including bullying experiences, social isolation metrics, attempt circumstances, lethality indices, and protective factors, would provide holistic risk factor profiles. Development and validation of culturally adapted personality screening instruments optimized for Indian populations, combined with cost-effectiveness analyses of personality-informed intervention strategies, would facilitate evidence-based resource allocation in resource-constrained settings.

## Conclusions

This comprehensive investigation of DSH patients in Kolar established Cluster B personality disorders, particularly borderline traits, as predominant among younger, female, socioeconomically disadvantaged, unmarried, unemployed individuals with recurrent suicide attempts, psychosocial stressors, and substance abuse. Psychiatric comorbidity, with adjustment disorders and depression as leading diagnoses, combined with a preference for liquid poison ingestion, reflects distinctive Indian sociocultural and environmental contexts. The documented personality-specific behavioral phenotypes - impulsive, emotion-dysregulated Cluster B patterns versus anxiety-driven, deliberative Cluster C characteristics - support multifactorial etiology requiring comprehensive biopsychosocial assessment and cluster-tailored therapeutic interventions. These findings underscore DSH as a complex public health challenge necessitating integrated prevention strategies spanning individual psychiatric treatment, family intervention, socioeconomic support, pesticide access restriction, and community mental health infrastructure development. Future research employing longitudinal methodologies with diverse populations will elucidate causal pathways and intervention effectiveness, ultimately reducing suicide mortality in vulnerable Indian populations.
